# Perception of Different Tone Contrasts at Sub-Lexical and Lexical Levels by Dutch Learners of Mandarin Chinese

**DOI:** 10.3389/fpsyg.2022.891756

**Published:** 2022-06-06

**Authors:** Ting Zou, Johanneke Caspers, Yiya Chen

**Affiliations:** ^1^School of English and International Studies, Beijing Foreign Studies University, Beijing, China; ^2^Leiden University Centre for Linguistics, Leiden, Netherlands; ^3^Leiden Institute for Brain and Cognition, Leiden, Netherlands

**Keywords:** acquisition of Mandarin tones, Dutch learners of Mandarin, L2 tone contrasts, sequence recall task, auditory lexical decision, developmental trajectory

## Abstract

This study explores the difficulties in distinguishing different lexical tone contrasts at both sub-lexical and lexical levels for beginning and advanced Dutch learners of Mandarin, using a sequence-recall task and an auditory lexical decision task. In both tasks, the Tone 2-Tone 3 contrast is most prone to errors for both groups of learners. A significant improvement in the advanced group was found for this tone contrast in the sub-lexical sequence recall task, but not in the lexical decision task. This is taken as evidence that utilizing tones in on-line spoken word recognition is more complex and demanding for L2 learners than in a memory-based task. The results of the lexical decision task also revealed that advanced learners have developed a stronger sensitivity to Tone 1 compared to the other three tones, with Tone 4 showing the least sensitivity. These findings suggest different levels of robustness and distinctiveness for the representation of different lexical tones in L2 learners’ lexicon and consequently different levels of proficiency in integrating tones for lexical processing. The observed patterns of difficulty are potentially related to the acoustic characteristics of different lexical tone contrasts as well as to the interference of the suprasegmental features of learner’s native language (i.e., the tonal contrasts of Dutch intonation) on the acquisition of the Mandarin lexical tone contrasts.

## Introduction

Considerable evidence indicates that tone presents a great challenge for adult non-tone language speakers learning Mandarin as a second language (L2). Most previous studies have examined phonemic processing of L2 tones, showing that not all tone contrasts are equally difficult to discriminate (e.g., Kiriloff, 1969; Wang et al., 1999). Distinguishing different tone contrasts in spoken word recognition, however, has been investigated less often (e.g., Pelzl et al., 2019; Ling and Grüter, 2022). Given that the link between phonemic and lexical processing is of key importance in L2 segment and tone acquisition (Wong and Perrachione, 2007; Schmitz et al., 2018), whether the confusable tone contrasts at the phonemic level also hinder lexical identification has remained an interesting issue to understand further. This study investigated the processing of tone contrasts at both sub-lexical and lexical levels in beginning and advanced learners of Mandarin, trying to reveal the relative difficulty in distinguishing different tone contrasts in lexical access and provide a more detailed developmental profile in L2 tone acquisition.

### Distinguishing Tone Contrasts in Native Mandarin Speakers

The tonal inventory of Mandarin Chinese consists of four lexically contrastive tones. The meaning of the syllable /ma/ can be “mother,” “hemp,” “horse,” and “to scold” when it is associated with Tone 1 (high-level), Tone 2 (mid-rising), Tone 3 (low-dipping), and Tone 4 (high-falling), respectively (Chao, 1956; Duanmu, 2000; Chen and Gussenhoven, 2008). Although other acoustic correlates, such as amplitude (Garding et al., 1986; Whalen and Xu, 1992) and vowel duration (Gandour and Harshman, 1978) also contribute to tone identification, the primary acoustic cue of tone perception is the fundamental frequency (F0) (Howie, 1976; Khouw and Ciocca, 2007). Among the tones, Tone 3 is often highlighted for having a high degree of variability. When produced in a pre-pausal position or in isolation, Tone 3 (T3) is realized with a dipping pitch contour. This tone has two variants in connected speech: it surfaces with a low falling pitch contour preceding Tone 1 (T1), Tone 2 (T2), Tone 4 (T4), or a neutral tone, and when preceding another T3, it is realized with a rising pitch contour like T2 (Yuan and Chen, 2014). Moreover, the underlying tone associated with a weak syllable is always described by the cover term neutral tone. Neutral tone has a static and mid target, but the target is realized with more pitch variability than lexical full tones: the pitch of a syllable with a neutral tone is substantially influenced by the tone in the preceding syllable (Chen and Xu, 2006).

In terms of the role of tonal information in lexical access and selection, some behavioral studies suggest that tone might be a weaker cue compared to segmental information, using tasks of speeded classification (Repp and Lin, 1990), vowel and tone monitoring (Ye and Connine, 1999), word reconstruction (Wiener and Turnbull, 2016) and priming (Sereno and Lee, 2015). However, more recent studies using online measures such as eye-tracking and event related potentials (ERPs) showed parallel processing of segments and tones in word recognition, arguing that the role of tonal information is comparable to that of segmental information (Schirmer et al., 2005; Malins and Joanisse, 2010, 2012; Zhao et al., 2011; Connell, 2017). It might be the case that the difference between tones and vowels is partly due to the difference in temporal availability of the cues. Prosody develops more slowly over time than segmental information, but every cue will be used in word recognition as soon as it is reliably perceived.

It is noteworthy that, for native Mandarin speakers, tone contrasts are not equally easy to distinguish and some tone contrasts are often poorly discriminated, which has been revealed in both L1 acquisition studies and tone perception research in adult Mandarin speakers. Some studies in L1 tone acquisition tested the discrimination of tone pairs at sub-lexical level. For instance, in an ERP study, Cheng et al. (2013) found that the contrast of T1 and T3 could elicit mismatch responses for both newborns and 6-month-old, but the T2-T3 contrast only caused mismatch responses for the older group, suggesting that the T2-T3 contrast could be more difficult and thus acquired in a later stage. Similar results were found in a tone discrimination task by Tsao (2008), in which Mandarin-learning infants (from 10 to 12 months of age) discriminated T1 and T3 more accurately compared to T2 and T3. Another line of research explored the lexical integration of tones using novel word learning and familiar word recognition tasks in L1 tone acquisition. Ma et al. (2017) tested 3-year-olds’ sensitivity to tone variation and found that they could use tones to learn new words, but T3 words were very difficult to learn compared to words with other tones. Using a preferential looking paradigm, Gao et al. (2011) examined the recognition of known words in toddlers (19–26 months) and found that the substitution between T2 and T3 could not be detected, while the other tonal substitution (T2 and T1) was detected with less difficulty. In a monosyllabic picture-pointing task, Wong et al. (2005) found that 3-year-olds were much less accurate in recognizing T3 (with an accuracy of 69%) relative to the other three tones (with accuracy rates higher than 80%) when discriminating tonal minimal pairs. Taken together, the convergent evidence from these studies suggests that the mastery of the Mandarin tone system is not uniform across the whole tonal inventory and the discrepancies could be observed even in native infants in terms of both the formation of phonetic categories and the integration of tones into word-level representations.

Even for adult native speakers with a mature phonological representation of the L1 tone inventory, their performance in tonal discrimination may also differ across different tone contrasts (e.g., Shen and Lin, 1991; Bent, 2005; Zhang et al., 2012; Li and Chen, 2015). For example, Bent (2005) found that for native Mandarin speakers, the accuracy of T2-T3 discrimination was lowest compared to other tone contrasts. The relatively long reaction time for the T2-T3 contrast in an odd-ball discrimination task in Zhang et al. (2012) also suggests a potential difficulty in distinguishing these tones.

To sum up, for both infant and adult native Mandarin speakers, T2 and T3 is the most confusable contrast in the process of category formation and spoken word recognition. The similar concave shapes in the f0 contours of the two tones may serve as an important source of the confusion. That is, in isolated form, a slight dipping exists in the contour of both T2 and T3 (Ho, 1976; Moore and Jongman, 1997; Fon and Chiang, 1999). Some perception studies confirmed that this acoustic similarity may serve as a perceptual cue in tone identification and thus can account for the confusion of this tonal contrast (Blicher et al., 1990; Shen and Lin, 1991; Gottfried and Suiter, 1997; Zou et al., 2012; Sun et al., 2018). Specifically, Gottfried and Suiter (1997) found that native Mandarin speakers were quite accurate in tone identification of intact syllables, but frequently misidentified T2 as T3 in syllables with only the initial part presented, due to the acoustic similarity of the initial portion of these two tones and the lack of perceptual cues present in their later rising contours. Shen and Lin (1991) revealed that the timing of the turning point and the degree of the fall (in the dipping contour) determine the identification of T2 and T3 and violation of the correlations of these acoustic features would lead to tone identification problems. Yet another potential reason for T2-T3 confusion may lie in the sandhi rule of T3—the contour of an initial T3 changes to a rising tone (just like T2) when followed by another T3. In the process of L1 acquisition, it has been found that this sandhi rule is acquired at a very early stage as the child begins to produce multi-word utterances (Li and Thompson, 1977). That is, for native speakers, T3 sandhi may help to establish a strong mental relation between T2 and T3 and thus makes this tone contrast difficult to distinguish (Huang and Johnson, 2010; Li and Chen, 2015; Pelzl, 2019).

### Distinguishing Tone Contrasts in Second Language Learners of Mandarin

Just like native Mandarin speakers, the acquisition of tones is also not uniform across the whole tonal inventory for L2 learners with a non-tonal L1. In prior research, L2 learners’ performance in tone perception has been widely studied at sub-lexical levels, focusing on tonal identification, categorization or discrimination in isolated syllables or disyllabic non-words (Kiriloff, 1969; Gottfried and Suiter, 1997; Wang et al., 1999; Guion and Pederson, 2007; Yang and Chan, 2010; Hao and de Jong, 2016; Shen and Froud, 2016, 2019; Zou et al., 2017; Hao, 2018a,b). In terms of tonal identification, Kiriloff (1969) found that among the four tones, there was a considerable number of incorrect identifications of T2 and a marked tendency to confuse T2 with T3 in beginning Australian English learners of Mandarin. In a training study, Wang et al. (1999) found that in both pre-test and post-test, T2 was the most difficult tone for English learners to identify, and T2-T3 was the most confusable pair. The T1-T4 pair was another problematic pair and most resistant to improvement. Hao (2012) found that for experienced English learners (with a mean study length of 2.68 years), T1 and T4 could be identified with high accuracy (around 90%), whereas tones T2 and T3 could not be correctly identified (with an accuracy rate of around 70%) and were mutually confusable. Gottfried and Suiter (1997) compared English learners’ identification of tones in intact syllables to syllables with varied portions removed and found that in all conditions, confusions were most common between T2 and T3. Regarding tonal discrimination, Hao (2018b) demonstrated that in an AXB task, T2-T3 was the most difficult pair with the lowest accuracy rate and longest reaction time for both beginning and more experienced English learners of Mandarin. Collectively, the difficulty in distinguishing T2 and T3 in isolated syllables and disyllabic non-words has been well-documented.

On the other hand, L2 learners’ performance in integrating tones in lexical representations has been investigated less often (Wiener et al., 2018; Pelzl et al., 2019, 2021a,b; Qin et al., 2019; Han and Tsukada, 2020; Ling and Grüter, 2022). The existing evidence of L2 lexical tonal processing generally suggests a persistent difficulty in tone processing. Pelzl et al. (2019) revealed that advanced L2 learners could achieve native-like performance in monosyllabic tone identification, but still encountered great difficulty in lexical tone processing, since they showed a very low accuracy in rejecting disyllabic tonal non-words in a lexical decision task. A subsequent study further illustrated that L2 learners’ overall accuracy was noticeably lower than the performance of native speakers in a disyllabic lexical decision task and L2 learners were significantly more likely to accept tone non-words incorrectly than vowel non-words (Pelzl et al., 2021a). The difficulty in utilizing tones for word recognition was also found in advanced Korean learners of Mandarin in a lexical decision task (Han and Tsukada, 2020). Moreover, this group of learners showed worst performance for the pair of T2–T3. Ling and Grüter (2022) further found that learners who performed better in the word recognition task also showed more categorical perception of tone in a tone identification task, indicating a link between phonemic and lexical processing.

In previous L2 perception studies, it has been demonstrated that even when L2 learners can distinguish non-native phonemic contrasts properly in low-level sub-lexical perception tasks, their performance often exhibited marked decline in more demanding lexical tasks. This is known as “graded learning” (Sebastián-Gallés and Díaz, 2012) in the acquisition of novel L2 sound contrasts. Examples of L2 segmental and suprasegmental graded learning include Japanese learners’ perception of the English /r-l/ contrast (e.g., Strange and Dittmann, 1984); native English listeners’ perception of non-English dental and retroflex stops (Werker and Tees, 1984; Werker and Logan, 1985) and French learners’ perception of Spanish lexical stress contrasts (Dupoux et al., 1997, 2001, 2008). These studies employ tasks tapping into different levels and modes of processing, ranging from sub-lexical discrimination to higher-level lexical processing utilizing abstract phonological representations. The consensus in their findings is that as the cognitive demands imposed by the task and stimuli increase, learners’ perception performance shows some significant decrement. Based on this line of findings, whether the difficulty in distinguishing tones at sub-lexical level would be different from distinguishing tone contrasts in real word recognition has become an important question since it may influence practical communication in L2.

The processing of tones in real word recognition has been tested in several studies by testing naïve non-native speakers of Mandarin (e.g., Wong et al., 2011). Using sound-to-word training paradigms which trains participants to associate members of minimal tone pairs with different meanings, these studies examined various factors that would potentially influence the training outcome, such as the contribution of individual variability in cue weighting (Chandrasekaran et al., 2010), the effect of individual musical experience (Wong and Perrachione, 2007), the influence of linguistic pitch processing ability (Bowles et al., 2016), the influence of learners’ L2 prosodic structures (Braun et al., 2014), the effect of the presence of an orthographic tone mark to the ability of associating tones with newly learned lexical items (Showalter and Hayes-Harb, 2013), as well as the influence of different designs of training paradigms (Perrachione et al., 2011). While the focus varies across these studies, the results lead to a convergent finding that with a proper amount and an appropriate approach of training, non-native speakers of Mandarin can gain significant improvement in utilizing tonal information for lexical identification. However, since these studies mainly tested naïve speakers’ training performance with a limited set of lexical items, their results cannot be generalized to represent real-life L2 learning processes. More work needs to be done concerning lexical tone processing in word recognition in experienced learners with a large vocabulary.

Additionally, the aforementioned studies (e.g., Pelzl et al., 2019; Ling and Grüter, 2022) on utilizing tones in word recognition mainly focused on comparing L2 learners’ performance in segmental processing versus tone processing. Less research, however, has been done to tap into the confusion patterns of different tone contrasts in real word recognition. Also, comparative research on the performance of learners with different Mandarin L2 proficiency levels has remained scarce, leaving open the detailed developmental profile of L2 learners’ integration of tones in lexical representation. To fill this knowledge gap, the current study sought to investigate L2 developmental patterns in distinguishing different tone pairs at both sub-lexical and lexical levels.

### The Present Study

In the present study, we would like to examine the performance of tone processing and the confusion patterns of different tone contrasts at both sub-lexical and lexical levels in beginning and advanced Dutch learners of Mandarin. Therefore, two experiments – a sub-lexical sequence recall task and a lexical decision task – were conducted and both beginning and advanced learners of Mandarin were recruited to explore the developmental path in lexical tone processing.

The sequence recall task followed the procedure in Dupoux et al. (2001, 2008) who have argued that this task provides a robust paradigm for testing the processing of novel L2 phonemic contrasts. In this task, participants were asked to learn to associate two disyllabic tonal minimal pairs with the keys “a” and “b” in a training phase with feedback. In the test phase, a sequence of non-words was presented, and the task for the participants was to transcribe the sequence in the correct order by typing a series of “a” and “b.” Phonetic variability was introduced in that the four non-words in a sequence are always produced by four different voices in random order.

The second task was an auditory lexical decision task, in which disyllabic Mandarin real words and non-words (i.e., with an incorrect tone on the first syllable) were used. Disyllabic real word-non-word pairs differing only in a consonant were used as a comparison condition. To tap into lexical processing, participants were asked to decide whether the presented word was a real word or not by pressing a key as soon as possible. Both accuracy rate and reaction time were recorded.

Since the acquisition of tone pairs for L2 learners was found to progress at different rates, for both tasks, all tone contrasts were tested in order to reveal the discrepancy in learners’ discrimination of different tone pairs. As suggested by previous studies, we expect the pair of T2 vs. T3 [and maybe T1 vs. T4, as suggested in Wang et al. (1999)] to be more difficult to discriminate than the other tone pairs.

## Experiment 1: Sequence Recall Task

### Participants

Twenty-six Dutch learners of Mandarin and fifteen Mandarin controls participated in the sequence recall experiment. All Dutch learners of Mandarin had received formal Chinese training from the Chinese Studies program at Leiden University. The beginning group consisted of six males and eight females (age: *M* = 20.83, SD = 2.82). Their Mandarin learning and speaking experience varied between 0.5 and 2 years (*M* = 1.21, SD = 0.51), and they had never lived in China. All the beginners had received formal instruction and they had metalinguistic skills like the ability to associate pitch contours with tone labels. The other fourteen participants (eight males and six females; age: *M* = 24.83, SD = 3.61) were advanced Mandarin learners, who had Mandarin experience between 3 and 14 years^1^ (*M* = 5.42, SD = 3.31), and had spent at least 1 year in China. Since for most participants, the score of a standard Chinese proficiency test (e.g., HSK test) was not available at the time of this experiment, we operationalized proficiency as years of study and experience of living in China. Both groups of learners had received formal language instruction and they had metalinguistic knowledge of lexical tones. They could associate pitch contours with tone labels and they were also instructed in their Chinese class about the contextualized form of T3: it should change to T2 before another T3, and become a low-falling tone (the so called half T3) before T1, T2, and T4. The native Mandarin control group consisted of three males and twelve females (age: *M* = 26.91, SD = 2.80). All were from the Northern part of China and could speak standard Mandarin. Since some tonal knowledge is necessary for participating in the sequence recall task, it was not possible to include a Dutch control group without any experience with lexical tone (see section “Procedure” for more details). Written informed consent to participate in the study has been obtained from all participants and all of them were paid for their participation.

### Materials and Design

All six possible tone pairs (T1-T2, T1-T3, T1-T4, T2-T3, T2-T4, and T3-T4) were tested in the experiment. In the experimental condition, three similar CVCV non-words (/pat*^h^*i/, /tik*^h^*a/, /kup*^h^*a/) were used. Each tone pair employed one non-word with the target tone on the initial syllable and a neutral tone on the final syllable (e.g., /pa^1^t*^h^*i/ - /pa^2^t*^h^*i/ for T1-T2). The vowel set of the non-words consisted of /a/, /i/, and /u/. In the consonant set, there are three voiceless pairs of stops (labial: /p/-/p*^h^*/; alveolar: /t/-/t*^h^*/; velar: /k/-/k*^h^*/). The resulting three non-words were combined with different tone pairs in a counterbalanced way (see Table 1). Two minimal pairs differing only in a consonant were used as the segmental control condition (/futa-fuka/; /supi-suti/). They were produced with T1 on the initial syllable and a neutral tone on the second syllable. The segmental control condition should not cause difficulty for all participant groups and so it was used as a baseline. The difficulty in tone processing can be revealed by comparison of the segmental control condition and the experimental condition. The stimuli were recorded four times by four native Mandarin speakers (two females and two males) from northern China. In addition, the word “OK” was recorded by a third female speaker. All items were recorded with a Sennheiser MKH416T microphone in the Leiden University Phonetics Lab using Adobe Audition (44.1 kHz, 16 bit). Mean duration of the stimuli was 637 ms.

**TABLE 1:** Non-word stimuli used in the sequence-recall task.

Experimental condition	Associated keys	
	A	B
T1-T2	/pa^1^t*^h^*i/	/pa^2^t*^h^*i/
T1-T3	/ti^1^k*^h^*a/	/ti^3^k*^h^*a/
T1-T4	/ku^1^p*^h^*a/	/ku^4^p*^h^*a/
T2-T3	/ku^2^p*^h^*a/	/ku^3^p*^h^*a/
T2-T4	/ti^2^k*^h^*a/	/ti^4^k*^h^*a/
T3-T4	/pa^3^t*^h^*i/	/pa^4^t*^h^*i/
Segmental control condition	/fu^1^ta/	/fu^1^ka/
	/su^1^pi/	/su^1^ti/

There are sixteen possible combinations for sequences of four non-words. To select the most difficult combinations, two Dutch learners of Mandarin and two native Mandarin listeners participated in a pilot with 192 stimuli (16 sequences × 2 repetitions × 6 tone pairs). It was found that participants make more errors for sequences with more variation in combinations. That is, the sequence of ABBA with one transition from A to B and another transition from B to A is more difficult than the sequence of AABB which only contains one transition from A to B. So, out of all sixteen possible sequences, the eight sequences with two and three transitions were selected (AABA, ABAA, ABBA, BAAB, BABB, BBAB, ABAB, BABA). In every sequence, the four non-words were produced by four different voices. The order of these four voices was counterbalanced between sequences. Each non-word was recorded four times by the four speakers. So, for each tone/segmental pair, all these tokens were used in the eight sequences. That is, for T1T2, sixteen tokens (4 voices × 4 tokens) of the non-word /pat*^h^*i/ were used. In total, we had 128 experimental trials (8 tonal/segmental pairs × 8 sequences × 2 repetitions).

### Procedure

Each participant was tested individually in the Leiden University phonetics lab with all 128 trials with the auditory stimuli presented through a Beyerdynamic DT-770 Pro headphone. The three groups of participants received instructions (in their native language) that they would learn some words. The six tone pairs were tested separately in six experimental blocks. To make sure the participants could associate the disyllabic tonal sequences with corresponding keys on the keyboard, each block consisted of a word learning phase, a training phase, and a main experimental phase.

In the learning phase, participants were instructed to press “a” on the keyboard to hear the first word, upon which a sound token of one non-word from a tone pair produced by a female speaker was played (e.g., /pa^1^t*^h^*i/). Then they were asked to press “b” on the keyboard, upon which the other sound token produced by the same female speaker was played (e.g., /pa^2^t*^h^*i/). After that, the participants were presented with “a” or “b” on the screen. Pressing the letter displayed on the screen led to the playing of one token of the corresponding non-word. In this way, participants heard all sixteen tokens that would be used in the training and experimental phases (4 voices × 2 members of the target tone pair × 2 repetitions) in random order by pressing the associated key.

In the subsequent training phase, participants received further training on the association between the non-words and their keys (i.e., “a” vs. “b”). They also learned the tone contrasts on the non-words. Their task was to identify the non-words. After hearing a non-word, the participants were asked to press its associated key (i.e., “a” or “b”). They got feedback on their choice as “Correct” or “Incorrect” on the screen. All sixteen tokens of the target tone pair were presented in random order. To make sure that participants became familiar with the tone contrasts and their corresponding keys, an accuracy rate of 80% was defined as the success criterion.

For the main experimental phase, only participants who reached the success criterion of 80% correct identification were invited. Therefore, for beginning learners, twelve (out of fourteen) participated in the main experiment. All advanced learners and native Mandarin listeners reached the criterion and took part in the main experiment.

We also tested a control group of monolingual Dutch speakers with the sequence recall task, but all three participants in our pilot study failed to reach the 80% correct criterion. For naïve listeners, this sequence-recall task with high phonetic variability turned out to be too difficult, which means that it was not feasible (given the time and resource constraints) to include a control group without any tonal experience in our experiment.

In the main experimental phase, there were two warm-up trials and sixteen experimental trials. In each trial the participants heard a sequence of four non-word tokens produced by four speakers and a following “OK” produced by a female voice. In order to lower the possibility of the participants translating the non-words into the associated letters immediately when listening to the stimuli, the inter stimulus interval among the four non-words was kept very short (50 ms) (cf. Dupoux et al., 2008). The “OK” following the non-word sequence was adopted to avoid the participant using echoic memory (Morton et al., 1971; Dupoux et al., 2008). The task for participants was to reproduce the order of the sequence by typing the associated keys as quickly and accurately as possible after hearing the word “OK.” After the response, the next trial started after a 1,500 ms pause.

The order of the six tonal blocks was randomized among participants. Within each block, the participants completed the word learning, the training, and the experimental phase (with the sequence-recall task). The control condition with two blocks of segmental minimal pairs was tested after the six tonal blocks. In total there were eight blocks. Each block took about 5 min to complete, and there was a 1-min break between blocks. The total experimental lasted about 40 min.

### Results

Analysis of the transcription results (i.e., correct or incorrect transcription of the non-word sequence) was performed with a mixed effects logistic regression model using R and the lme4 package (Bates et al., 2014). For all trials, a model was constructed with Participant Group (i.e., native Mandarin listeners, beginning Dutch learners and advanced Dutch learners), Tone Pair (i.e., six tone pairs and one segmental control condition) and their interaction as fixed effects (fixed effects are indicated with capital initial letters). Intercepts for participants and items were added as random effects. Treatment coding was used in this model. *Post hoc* comparisons of differences between different levels within each effect were conducted using Multcomp package with Bonferroni adjustment in R (Hothorn et al., 2008).

The statistical results for the model of response accuracy are presented in Table 2. The χ^2^ and corresponding *p-*values for fixed and random effects were obtained from likelihood ratio tests. There was a significant effect of Participant Group, Tone Pair as well as a significant interaction between Participant Group and Tone Pair. Below, we will present a more detailed *post hoc* analysis of the interaction of Participant Group and Tone Pair.

**TABLE 2:** Summary of a mixed-effect logistic model for response accuracy.

Fixed effects	Accuracy		
	df	χ^2^	*p*
Participant group	2	3.67	<0.001
Tone pair	6	140.14	<0.001
Participant group × Tone pair	12	116.60	<0.001
Random effects			
1| Participant		287.59	<0.001
1| Item		38.90	<0.001

**FIGURE 1:**
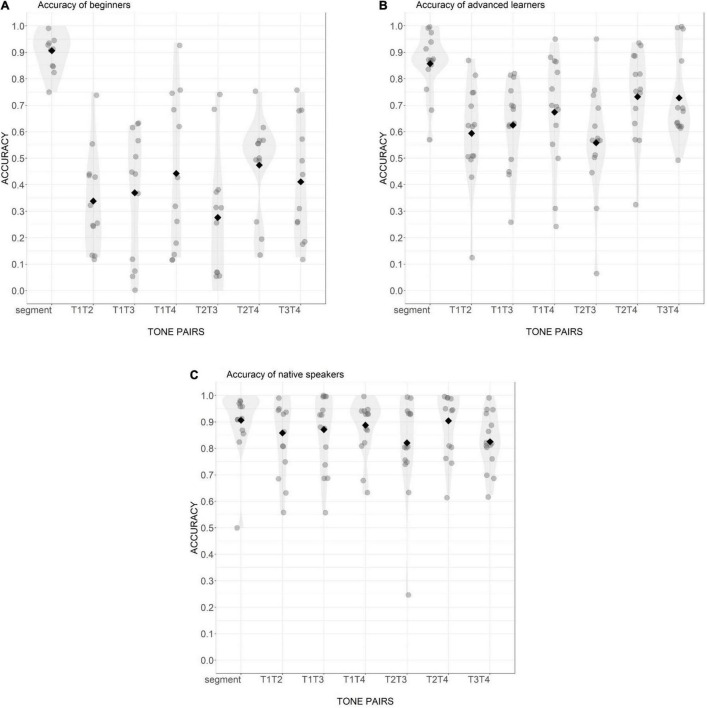
Distribution of sequence-recall accuracy of beginning learners **(A)**, advanced learners **(B)**, and native Mandarin speakers **(C)** in six tone pairs and the segmental condition. Gray points are individual participant means. Black diamonds are condition means.

The sequence recall accuracy of the six tone pairs and the segmental control condition for the three groups is presented in Figure 1. In the control condition, the overall accuracy was high across all three participant groups with no statistical difference among groups (BL = 90.6%, AL = 85.7%, NM = 90.6%; all *p*-values > 0.05). This indicates that all three groups can process segmental contrasts with little difficulty. It also means that their phonological working memory ability enabled them to perform properly in this task.

In the tonal conditions, pairwise comparison demonstrated that the accuracy was significantly different between each two groups of participants for all tone pairs except for T3T4 (all *p*-values < 0.05). Specifically, in all tone pairs, the accuracy of beginning learners was low, but still high above chance performance level (chance level for 4-word sequence equals 1/2^4^, which is 6%). Compared to beginning learners, the advanced learners had significantly higher accuracy in tone processing, but their performance was still below that of the native Mandarin listeners. Only for the pair of T3T4, the difference between advanced learners and native speakers failed to reach statistical significance (AL = 72.8%, NM = 82.5%; Est. = 0.77, *z* = 2.10, *p* > 0.05).

For the native Mandarin speakers, the performance in tonal and segmental conditions was comparable in general. Pairwise comparison showed that the accuracy of segmental pairs was only significantly higher than the accuracy of T2T3 (*z* = −4.13, *p* < 0.001), which among all tone pairs, showed the lowest accuracy (78.8%), significantly lower than T2T4 (90.4%) which was the most accurate pair (Est. = 1.09, *z* = 3.38, *p* < 0.05). The accuracy rate was comparable among all other tone pairs.

For beginning learners, the accuracy in the segmental condition was significantly higher than that of all tone pairs (all *p*-values < 0.001). Among tone pairs, the most difficult was T2T3 (27.0% correct), followed by T1T2 (33.9%), T1T3 (37.0%), T3T4 (41.1%), T1T4 (44.3%), and T2T4 (47.4%). *Post hoc* analyses reveal that the accuracy of T2T3 was significantly lower than that of T1T4 (Est. = −0.83, *z* = −3.05, *p* < 0.05) and T2T4 (Est. = 0.97, *z* = 3.58, *p* < 0.01). The accuracies of other tone pairs were not significantly different from each other.

Like beginning learners, advanced learners were significantly more accurate in the segmental condition than in all tone pairs (all *p* < 0.02). Within the tonal conditions, T2T3 (55.8%) was again the most difficult pair, followed by T1T2 (59.4%), T1T3 (62.5%), T1T4 (67.4%), T3T4 (72.8%), and T2T4 (73.2%). The accuracy of T2T3 was significantly lower than T3T4 (Est. = 0.82, *z* = 3.24, *p* < 0.05) and T2T4 (Est. = 0.85, *z* = 3.34, *p* < 0.05). But overall, advanced learners performed more accurately than beginners.

## Experiment 2: Lexical Decision Task

### Materials and Design

Ten disyllabic word-non-word pairs were chosen for each tone pair. For Dutch listeners, a stimulus that ends with T1, T2, or T3 can potentially be interpreted as carrying a non-final boundary tone (H%), which signals either continuation or question. T4 sounds like a final fall (H*L L%) (Gussenhoven, 2005; Braun and Johnson, 2011). To avoid the potential influence of different boundary tones from the listeners’ L1, we kept the tone on the second syllable constant throughout the entire experiment, only using real words with T1. Moreover, the effect of tonal coarticulation on the realization of the tonal contour on the first syllable has also been considered. In Mandarin, the magnitude of carryover tonal coarticulation is much larger compared to the anticipatory coarticulation. That is, in a disyllabic word, “the final portion of the first tone closely follows its intended trajectory to the end of the syllable” (Xu, 1997), while the contour second tone shows more deviation and only approaches its target in the later portion of the syllable. This predominance of carryover coarticulation may result from the interaction of physiological constraints and perceptually motivated constraints that require tonal target realization. As suggested by Flemming (2011), the stronger assimilatory carryover effect (compared to the anticipatory effect) is shaped by the greater importance on the constraint to realize tonal targets over syllable rhymes (rather than onsets), as tones are more perceptible and easier to identify when realized during rhymes which have high intensity periodicity and rich harmonic structure. Therefore, the non-words were constructed by changing the tone on the first syllable to reduce the influence of tonal coarticulation.

The non-words were constructed by changing the tone on the first syllable of the real words. That is, real words and non-words minimally contrast in the tone on the first syllable. Tone pairs were tested bi-directionally, which means that there were twelve pairs in total (T1-to-T2, T2-to-T1, T1-to-T3, T3-to-T1, T1-to-T4, T4-to-T1, T2-to-T3, T3-to-T2, T2-to-T4, T4-to-T2, T3-to-T4, and T4-to-T3). For the tone pair T1-to-T2, ten real words with T1 on the first syllable were selected, and the corresponding non-words were constructed by changing T1 to T2 on the first syllable, while the tone of the second syllable was kept constant (i.e., T1). For example, the corresponding non-word for the real word 春天 /tş^h^uən^1^ t*^h^*iεn^1^/ (spring) was /tş*^h^*uən^2^ t*^h^*iεn^1^/. As a comparison condition, another 40 disyllabic word-non-word pairs which differed only in the initial consonant of the first syllable were chosen. There were ten words with T1 on the initial syllable, and ten words each with T2, T3, and T4. The second syllable always carried T2. The non-words were constructed by changing the manner of articulation of the initial consonant in the initial syllable. For instance, the corresponding non-word for the real word 公园 /kuŋ^1^yεn^2^/ (park) was /k*^h^*uŋ^1^yεn^2^/. All these consonant contrasts were familiar to Dutch listeners. Although /k/ and /k*^h^*/ is not a possible phoneme pair in Dutch, the listeners must have learned these phonemes as part of the English and Mandarin sound systems.

Vocabulary size can play an important role in L2 speech perception. To make sure that all participants were familiar with the stimuli, the real words were selected from the first-year text books of the Chinese studies program at Leiden University. However, the words are still not equally familiar to the learners (esp. for the beginners). The list of stimuli and the hit rate (the percentage of correct identification of real words) of both beginners and advanced learners for each word has been presented in the Supplementary Material.

Non-word type has also been reported to influence the wordlikeness judgment in Mandarin. It has been shown that non-words with phonotactic violations (e.g., with a consonant cluster which is illegal in Mandarin) can be easily and correctly identified, whereas non-words which do not violate phonotactics but form a regular segment-tone combination gap (e.g., /dai2/) could not be easily and quickly ruled out by native speakers (Wiener and Turnbull, 2016). To maintain a similar wordlikeness level across non-words, only phonotactically legal syllables were used when constructing non-words in this experiment. To make sure all the non-words could be correctly recognized as non-words by native speakers, a pre-test was conducted in which ten native speakers were asked to rate the wordlikeness of the non-words using a five-point scale with point 1 referring to real word and point 5 for non-word. The average point for all the non-words used in our task was 4.56.

Word frequency and lexical neighborhood density can affect the RT of lexical decision. Phonological neighborhood density refers to the similarity of the target words to other words in the mental lexicon, defined by the number of phonologically similar neighbors. Past research reported longer RTs for high-density words but shorter RTs for high-frequency words. The frequency effect was more salient for low-neighborhood density (Goh et al., 2009). So, these two factors were also carefully controlled in this experiment so that the overall word frequency, as computed with SUBTLEX-CH (Cai and Brysbaert, 2010) and analyzed with a one-way ANOVA, did not differ significantly across tone pairs and the segmental condition (The mean of the log frequency across all pairs is 3.03 and the standard deviation is 0.05) [*F*_(12,147)_ = 0.04, *p* > 0.99]. The neighborhood density of the first and second syllable of the disyllabic words was computed as the number of homophones according to the Modern Chinese Dictionary. The mean neighborhood density of the first syllable across all pairs was 11.89 and standard deviation was 2.13, the mean and standard deviation of the second syllable was 13.47 and 3.04. Statistical results showed that neighborhood density was also not significantly different across tone pairs and the segmental condition [first syllable: *F*_(12,147)_ = 0.57, *p* = 0.87; second syllable: *F*_(12,147)_ = 0.80, *p* = 0.65].

All stimuli were recorded by a female native Mandarin speaker who was born and brought up in Beijing with a normal speech rate. The recording was conducted with a Sennheiser MKH416T microphone in the Leiden University Phonetics Lab using Adobe Audition (44.1 kHz, 16 bit). The average duration of all stimuli was 774 ms. For each tone pair, ten real words and ten non-words were used. In total, we had 320 experimental trials: 12 tone pairs × 10 word-non-word pairs × 2 word types (real word/non-word) + 4 segmental conditions × 10 word-non-word pairs × 2 word types (real word/non-word).

### Procedure

The same groups of participants as in Experiment 1 were tested. All three groups received instructions in their native language. They were asked to decide whether the word they heard was a real word in Mandarin or not as quickly as possible by pressing the button “1” (for real word) or “2” (for nonce word), respectively, on the keyboard. The participants were informed that the non-words were very similar to real words but with a difference in tone or initial consonant on the first syllable. The order of the 320 stimuli was randomized for each participant. Before the real test, there was a warm-up session, in which two pairs of word/non-words differing in the initial consonant of the first syllable were presented, to help the participants get familiar with the associated buttons (1 vs. 2). None of these words was used in the main experiment. During the warm-up phase, the participants received a “Correct” or “Incorrect” message on the screen as feedback. The main experiment consisted of four blocks of 80 trials. Each trial began with the presentation of a fixation cross on the screen for 500 ms. The stimulus was presented 500 ms after the disappearance of the cross. After the participant’s response, there was no longer feedback and the next trial started after a 1,500 ms pause. The total experimental lasted about 30 min.

### Results

The response to each trial was classified as a hit (H) (correctly recognizing a real word), a false alarm (F) (mistakenly classifying a non-word as real word), a miss (failing to recognize a real word), or a correct rejection (correctly rejecting a non-word). For each participant, an A′ (A prime) score was calculated for each tone pair across items with the formula $A^{\prime }=0.5+\left[sign\left(H-F\right)\frac{{\left(H-F\right)}^{2}+\left|H-F\right|}{4\max\left(H,F\right)-4HF}\right]$ (Stanislaw and Todorov, 1999). A′ is a bias-free estimate of sensitivity to word-non-word classification, which takes account of both hit rate and false-alarm rate. The range of an A′ score is from 0.5, which indicates real words cannot be distinguished from non-words, to 1, which suggests perfect performance in word-non-word classification (Stanislaw and Todorov, 1999; Macmillan and Creelman, 2004).

Analyses of A′ scores were performed with a linear mixed-effects model using R and the lme4 package (Bates et al., 2014). A model was constructed with Participant Group (i.e., native Mandarin listeners, beginning Dutch learners, and advanced Dutch learners), Word Pairs (i.e., twelve tone pairs and four segmental conditions) and their interaction as fixed effects. Intercepts for Participant was used as random effect. Treatment coding was used for this model.

The raw reaction times for correct responses was converted to logarithmic RT to achieve better normalcy. The analysis of log RT was also performed with a linear mixed effect model using R and the lme4 package (Bates et al., 2014). A model was constructed with Participant Group, Tone Pairs, and their interaction as fixed effects (fixed factors are indicated with capital initial letters). Intercepts for participants as well as by-participant random slope for the effect of participant group were entered as random effects. Treatment coding was used for this model.

**FIGURE 2:**
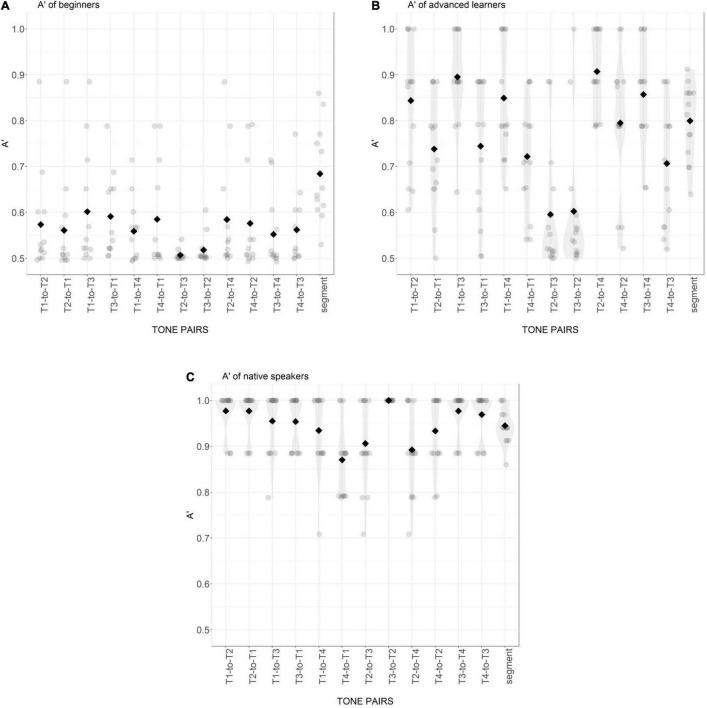
Distribution of A′ score of beginning learners **(A)**, advanced learners **(B)**, and native Mandarin speakers **(C)** for 12 tone pairs and the segmental condition. Gray points are individual participant means. Black diamonds are condition means.

**FIGURE 3:**
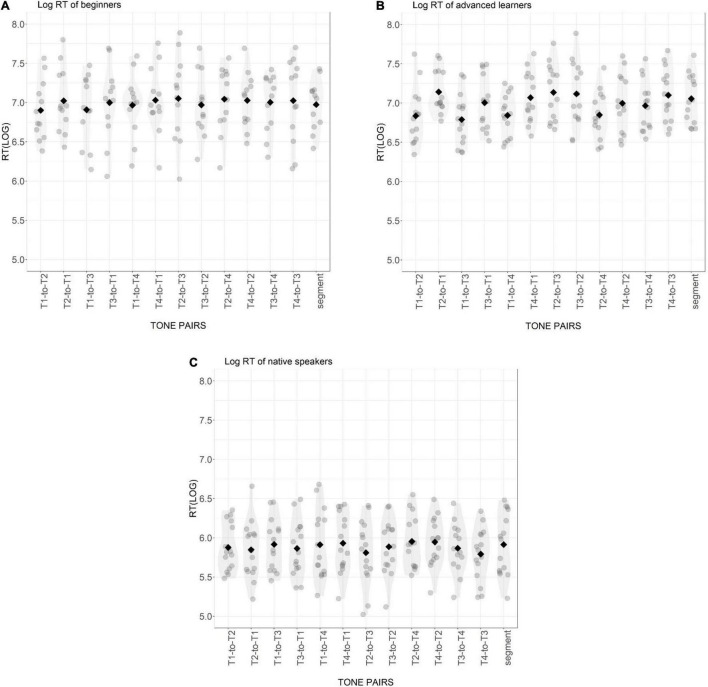
Distribution of log-transformed reaction time of beginning learners **(A)**, advanced learners **(B)**, and native Mandarin speakers **(C)** for 12 tone pairs and the segmental condition. Gray points are individual participant means. Black diamonds are condition means.

For both models of accuracy and reaction time, post-hoc comparisons of differences between different levels within each effect were conducted using the Multcomp package with Bonferroni adjustment in R (Hothorn et al., 2008). The average A′ scores for each participant group are shown in Figure 2. The log-transformed RTs and the statistical results for the three groups in different conditions are presented in Figure 3 and Table 3.

**TABLE 3:** Summary of mixed effects models for A′ score and RT.

Fixed effects	A′ score			RT (log)		
	df	χ^2^	*p*	df	χ^2^	*p*
Participant group	2	76.12	<0.001	2	54.72	<0.001
Tone pair	12	102.78	<0.001	12	12.64	n.s.
Participant group × Tone pair	24	174.4	<0.001	24	101.58	<0.001
Random effects						
1| Participant		507.38	<0.001		2381	<0.001
1| Item					847.64	<0.001

For A′ scores (see Table 3), there was a significant main effect of Participant Group and Tone Pair, and a significant interaction. For RT, there was a significant main effect of Participant Group and a significant interaction between Participant Group and Tone Pair. The effect of Tone Pair was not significant for RT.

In the segmental control condition, the A′ scores of all three groups significantly differed from each other (all *p*-values < 0.5). Specifically, the advanced learners showed a significant improvement compared with the beginning learners, but still did not perform like native Mandarins (see Figure 2). The mean RTs of beginner and advanced learners did not differ from each other in the control condition, but both learner groups responded significantly more slowly than native Mandarin listeners (BL vs. NM: Est. = −1.08, *z* = −8.03, *p* < 0.001; AL vs. NM: Est. = −1.15, *z* = −8.94, *p* < 0.001) (see Figure 3).

In the tonal condition, the three groups demonstrated a similar pattern in A′ scores compared to that in the segmental control condition, with the native group showing the highest scores, the beginning learners the lowest, and the advanced learners in between. The advanced learners showed a significant improvement in most tone pairs compared to the beginning learners (all *p*-values < 0.002), except for the pairs of T2-to-T3 and T3-to-T2, indicating that the sensitivity to T2 and T3 was still very low and resistant to improvement. The A′ scores of the native Mandarins were significantly higher than those of the advanced learners in most tone pairs (all *p*-values < 0.001), except for the pair of T2-to-T4 (Est. = −0.01, *z* = −0.39, *p* = 1), T1-to-T3 (Est. = 0.06, *z* = 1.58, *p* = 0.343) and T1-to-T4 (Est. = 0.09, *z* = 2.24, *p* = 0.075). For RT, both learner groups responded significantly more slowly than the native Mandarins in all tone pairs (all *p*-values < 0.001). The RTs of the two learner groups did not show significant difference (all *p*-values > 0.05).

Native Mandarin listeners showed high sensitivity in both the segmental control and the tonal condition, and there was no significant difference between these two conditions in both A′ scores and RT. Within the tonal condition, the overall A′ score was high across tone pairs. Among tone pairs, T4-to-T1 was the one with the lowest sensitivity score, significantly lower than that of T3-to-T2, T1-to-T2, T2-to-T1, T3-to-T4 and T4-to-T3 (all *p*-values < 0.05). T3-to-T2 was the one with the highest sensitivity score, significantly higher than that of T4-to-T1 (Est. = −0.13, *z* = −4.391, *p* < 0.001), T2-to-T3 (Est. = 0.11, *z* = 3.80, *p* < 0.5) and T2-to-T4 (Est. = 0.11, *z* = 3.64, *p* < 0.05). For each two tones, only T2 and T3 showed directional difference in sensitivity with A′ scores for T3-to-T2 significantly higher than for T2-to-T3. This suggests that when T3 was produced as T2, native Mandarin listeners were more likely to make a correct response than the other way round. In the initial position (before a T1 in the following position), the category of T3 was better-established than T2. For native Mandarin listeners, the RT was not significantly different across tone pairs.

For beginning learners, their A′ score in the segmental control condition was on average higher than in the tone condition, confirmed by pairwise comparisons of the A′ score in the segmental condition against that in the tone pairs T2-to-T3, T3-to-T2, T1-to-T4, T2-to-T1, T3-to-T4 and T4-to-T3 (all *p*-values < 0.05). Across tone pairs, comparable patterns were observed in terms of both the A′ score and RT.

For advanced learners, the A′ score of the segmental control condition was comparable to the score for most pairs in the tone condition, only significantly higher than that of T2-to-T3 (Est. = −0.20, *z* = −6.68, *p* < 0.001), T3-to-T2 (Est. = −0.20, *z* = −6.45, *p* < 0.01) and significantly lower than T2-to-T4 (Est. = 0.11, *z* = 3.51, *p* < 0.05). The RT of the segmental condition was shorter than that of tone pair T1-to-T3 (Est. = −0.27, *z* = −3.657, *p* < 0.05). Across the tone pairs, *post hoc* tests demonstrated that the A′ scores of T2-to-T3 and T3-to-T2 were significantly lower than of other tone pairs (all *p*-values < 0.01). The RT of T2-to-T3 was significantly longer than T1-to-T2 (Est. = 0.34, *z* = 3.58, *p* < 0.05), T1-to-T3 (Est. = 0.39, *z* = 4.09, *p* < 0.01), T1-to-T4 (Est. = 0.33, *z* = 3.49, *p* < 0.05) and T2-to-T4 (Est. = −0.34, *z* = −3.53 *p* < 0.05). The response to T3-to-T2 was significantly slower than the response to T1-to-T2 (Est. = 0.33, *z* = 3.45, *p* < 0.05) and T1-to-T3 (Est. = 0.38, *z* = 3.97, *p* < 0.01). These patterns suggest that compared to beginning learners, sensitivity to tone information in lexical access was better for advanced learners, but this improvement was not equal across tone pairs, with the confusion between T2 and T3 most resistant. The sensitivity scores of T2-to-T3 and T3-to-T2 were comparable, indicating that these two tones were mutually confusable, but the symmetry did not hold for other tone pairs. There was a significant difference in the A′ score between T1-to-T2 and T2-to-T1 (Est. = −0.11, *z* = −3.45, *p* < 0.05), T1-to-T4 and T4-to-T1 (Est. = −0.13, *z* = −4.17, *p* < 0.01) as well as between T1-to-T3 and T3-to-T1 (Est. = −0.15, *z* = −4.93, *p* < 0.01), suggesting that it was easier for advanced learners to correctly recognize real words with T1, and to correctly reject non-words with T1 substituted by T2, T3 or T4 than vice versa. There was a similar asymmetry with T4 evident in the significant differences between A′ scores of T2-to-T4 and T4-to-T2 (Est. = −0.11, *z* = −3.66, *p* < 0.05), T3-to-T4 and T4-to-T3 (Est. = −0.15, *z* = −4.919, *p* < 0.01), as well as T1-to-T4 and T4-to-T1 (Est. = −0.13, *z* = 4.17, *p* < 0.01). It was more difficult for advanced learners to make a correct response when T4 was substituted by another tone than the other way round. A trend of shorter RTs was also observed in T1-to-T2, T1-to-T3, and T1-to-T4 than to T2-to-T1, T3-to-T1 and T4-to-T1, respectively, without reaching statistical significance. It is worth noting that these asymmetric patterns for T1 vs. other tones and T4 vs. other tones were only found for advanced learners.

## Discussion

In the present study, we investigated the sensitivity to and utilization of lexical tones in both sub-lexical and lexical processing by Dutch learners of Mandarin, with a sequence recall task and a lexical decision task. Generally speaking, in the sequence recall task, the advanced learners exhibited a significant better performance compared to beginners. In the lexical decision task, advanced learners also performed significantly better in correctly identifying real words and rejecting non-words which were minimally different from real words in tones. These results suggest that language learning experience facilitates the forming of new tonal categories, and tonal information can be integrated in lexical representation by experienced learners.

In both tasks, however, we also observed that the performance of the advanced learners was still significantly lower than that of native speakers. Moreover, the RTs were much longer for advanced learners than for native Mandarin listeners in all conditions in the lexical decision task. We take this as evidence that their lexical representations of tones are still in development; the encoding of lexical tones is not as robust and distinctive as that of native Mandarin listeners and consequently, the utilization of tones for lexical processing is not yet automatic and accurate, which is compatible with the findings of Pelzl et al. (2019).

Importantly, our results also revealed differences in distinguishing different tone contrasts at sub-lexical and lexical levels. In the sub-lexical sequence recall task, the tone pair of T2 and T3 remained the most difficult contrast for both groups of learners, although the advanced learners improved significantly compared to the beginners. In the lexical decision task, these two tones were mutually confusable for both groups of learners and proved resistant to improvement. Burnham (1986) proposed that phonetic contrasts can vary on a continuum from “fragile” to “robust,” and the salience of the phonetic contrast can be used to account for the relative difficulty for naïve non-native listeners and L2 learners in their discrimination of non-native contrasts. Psycho-acoustically salient contrasts are learned at a very early stage in L1 acquisition and can be perceived with ease by non-native listeners. Less salient contrasts, however, are developed later in L1 acquisition and are also difficult to learn for L2 learners. The contrast of T2 and T3 in the current study is clearly a case of these less salient contrasts. Despite that the advanced learners have progressed significantly compared to beginners in discriminating T2 and T3 in a cognitively demanding sequence-recall task, their performance in utilizing this contrast to identify real word is still as poor as beginners, indicating different levels of acquisition of a tonal system. That is, being able to differentiate a tonal contrast does not automatically lead to a robust and precise representation of the tones in learners’ lexical memory. It takes a longer time for learners to learn such a “fragile” contrast and utilize the tonal information for lexical processing.

Interestingly, we also observed that, for some tones, there are uni-directional perceptual difficulties for L2 speakers. Specifically, in the lexical decision experiment, advanced learners were significantly more accurate in recognizing real words with T1 and rejecting non-words in which T1 was produced as one of the other three tones than the other way round, which suggests that the category of T1 had been relatively well established when compared to the other three tones. On the other hand, they gave less correct identification when T4 was produced as T1, T2, or T3, suggesting that the category of T4 was relatively less well-established when compared to the other three tones in pairs. One possible explanation is that all words we used in the lexical decision task have T1 in the second syllable, and the encoding and decoding of T1 may have benefited from the repeated exposure of the correct usage of T1 in this task. The similarity between subsequent syllables in words with T1T1 combination may also help with the correct identification of T1 on the first syllable. More likely, our results are related to two aspects of the prosodic features of the learners’ native language. The first concerns the perceptual dimensions of pitch movements. Gandour (1983) investigated the perceptual dimension of lexical tones and the influence of different language backgrounds on tonal perception. His results suggest that compared to tone-language speakers, English speakers are more sensitive to pitch height than to pitch direction, which has been confirmed by later findings in e.g., Maddox et al. (2013) and Chandrasekaran et al. (2016). In particular, Hao (2018a) showed that English speakers are very sensitive to the F0 onset in the identification of T1. Given the similar functions and characteristics of pitch movements in English and Dutch, it is possible that the excellent performance (i.e., both the identification of correct T1 and rejection of incorrect T1) in the lexical decision task with T1 (a high-level tone)-words by our advanced Dutch learners of Mandarin may in part be due to their enhanced sensitivity to pitch height.

The second, in relation to the failed detection of a mispronounced T4 (when replaced by the other three tones), concerns the role of tonal categories in the intonation system of Dutch. Previous studies have indicated that the acquisition of L2 sound categories can be influenced by learners’ L1 sound systems. In a series of cross-linguistic studies, So and Best (2010, 2014) asked native English and French speakers to match Mandarin tones with the given intonation categories in their L1s (“statement,” “question,” “flat-pitch,” and “exclamation”). The results suggested that tone categories can be assimilated into listeners’ L1 prosodic systems to some extent, however, there did not seem to be a simple one-to-one mapping pattern between lexical tones and intonation categories. For Dutch learners of Mandarin, the pitch fall in T4 is similar to the falling pitch accent in Dutch, which may be considered the most common form of pitch accent in Dutch (Gussenhoven, 2005). This similarity may make T4 less marked for Dutch learners, and consequently, the contrasts of T4 with other lexical tones become weakened.

Besides the perceptual difficulty and L1 prosodic interference, another potential source of difficulty in the lexical decision task is that learners may forget the tones of the disyllabic words or did not establish the specific syllable-tone association when the word was learned. In that way, it will be hard for them to reject non-words with wrong tones. According to information-theoretic methods (Garner and Miller, 1988; Tong et al., 2008), in the context of word recognition, the ability of a given signal to constrain recognition is related to its probability of occurring in a corresponding communication system. Due to the small tone inventory compared to that of the segments, each tone occurs more frequently and associates with more words in Mandarin Chinese than consonants and rimes, which makes tone less informative and poorer at constraining word recognition (Tong et al., 2008; Zou et al., 2021). Therefore, L2 learners may make less effort on the learning of this unfamiliar suprasegmental contrast which is less informative in constraining word identification compared to segments. In addition, the frequency of segment plus tone combination in learners’ experience also plays an important role in tone processing. It has been found that learners were better at recognizing new words that were homophonous with previously learned words (Liu and Wiener, 2020, 2021) and it could be the case that learners may be more automatic in tone processing with frequently used words. This factor still needs to be examined in future studies.

For native speakers, in the sequence recall task, T2-T3 was the most difficult pair with the lowest accuracy. In the lexical decision task, although the A′ scores were very high across all tone pairs for native Mandarin listeners, there was an asymmetry in accuracy for the contrast of T2 and T3. Native speakers performed better in recognizing real words with T3 and rejecting non-words in which T3 was produced as T2 than vice versa. This suggests that the category of T3 (a low tone) in word initial position is more robustly and less ambiguously encoded compared to T2 (a rising tone), likely due to the fact that an initial low falling pitch contour (as the realization of T3 before T1) can only be perceived as T3 by native listeners, but an initial rising pitch contour could be attributed to two different lexical tones: T2 or the sandhi form of T3 (According to the tone sandhi rule, T3 becomes a rising tone which sounds like T2 when followed by another T3; see Yuan and Chen (2014) for further details and references therein). This may hinder the participants in making correct responses when T2 is followed by another syllable. These results are also in line with findings on the neural activation of T2 and T3 words in native Mandarin speakers (Li and Chen, 2015). Further experiments with more participants and stimuli can help to consolidate the findings.

It should be noted that, finding L2 Mandarin learners for lab-based studies is a major challenge, especially advanced learners. Given that the levels for tone and segmental pairs were 6 and 13 for the two experiments, the sample size of the current study was small. Although the main findings of the current study fit coherently into the literature, further research with larger samples of participants is certainly needed to confirm current findings.

## Conclusion

With a sequence recall task and an auditory lexical decision task, this study explored the patterns and levels of difficulty in distinguishing and utilizing lexical tones during sub-lexical and lexical processing for non-tone language learners. In the sub-lexical sequence-recall task, significant improvement for advanced Dutch learners of Mandarin was observed in all lexical tone pairs compared to beginners. In the lexical decision task, T2 and T3 was mutually confusable for L2 learners, but no significant improvement was found between beginners and advanced learners, indicating that it does take a long process to acquire such a difficult phonemic contrast which is also difficult for native speakers in L1 acquisition. During the process of tone learning, advanced learners gradually developed a stronger sensitivity to T1; the establishment of the T4 category, on the other hand, seems less robust, resulting in the least sensitivity that learners showed for T4 replacement (by other lexical tones). The perceptual difficulty of T4 for Dutch learners can be attributed to the interference from L1 suprasegmental features (i.e., the falling contour in Dutch intonation) on the acquisition of the lexical tone contrasts in Mandarin. Results of our study thus make a unique contribution to the growing body of literature aimed to understand the role of second language learning experience and native language sound system in the developmental trajectory of L2 acquisition, as well as the role of different levels of proficiency in distinguishing and utilizing newly learned sound category contrast for different levels of L2 speech processing.

## Data Availability Statement

The raw data supporting the conclusions of this article will be made available by the authors, without undue reservation.

## Ethics Statement

The studies are part of the language research carried out at the Leiden University Center for Linguistics, which is reviewed and approved by the Commissie Wetenschappelijke Integriteit at Leiden University. The participants provided their written informed consent to participate in this study.

## Author Contributions

TZ designed and carried out the experiments, analyzed the data, and drafted the first version of the manuscript. YC and JC made substantial contributions to the design of the study, interpretation of the data, and drafting of the final manuscript. All authors provided critical feedback and helped to shape the research.

## Conflict of Interest

The authors declare that the research was conducted in the absence of any commercial or financial relationships that could be construed as a potential conflict of interest.

## Publisher’s Note

All claims expressed in this article are solely those of the authors and do not necessarily represent those of their affiliated organizations, or those of the publisher, the editors and the reviewers. Any product that may be evaluated in this article, or claim that may be made by its manufacturer, is not guaranteed or endorsed by the publisher.

## Footnotes

1 Within the advanced group, the majority of participants had Mandarin experience of 3–5 years and only two (out of 14) had longer experience (12 and 14 years).
