# Supplementary material for: Perception of Different Tone Contrasts at Sub-Lexical and Lexical Levels by Dutch Learners of Mandarin Chinese
Source: Front Psychol. 2022 Jun 6;13:891756. doi: 10.3389/fpsyg.2022.891756 (PMC9207513; doi:10.3389/fpsyg.2022.891756)
Supplement: Supplementary file 1 [file Data_Sheet_1.PDF]

Stimuli used in the lexical decision task.

T1-to-T2					
Real Words			Beginners' Hit Rate	Advanced Learners' Hit Rate	Non-words
餐厅	can1ting1	*restaurant*	0.92	0.93	*can2ting1
出发	chu1fa1	*set out*	0.75	0.79	*chu2fa1
出租	chu1zu1	*rent*	0.67	0.93	*chu2zu1
春天	chun1tian1	*spring*	0.92	1.00	*chun2tian1
发音	fa1yin1	*pronunciation*	1.00	0.93	*fa2yin1
秋天	qiu1tian1	*autumn*	0.58	0.79	*qiu2tian1
声音	sheng1yin1	*sound*	0.83	1.00	*sheng2yin1
听说	ting1shuo1	*heard about*	1.00	1.00	*ting2shuo1
西瓜	xi1gua1	*water mellon*	1.00	1.00	*xi2gua1
应该	ying1gai1	*should*	0.92	0.93	*ying2gai1

T2-to-T1					
Real Words			Beginners' Hit Rate	Advanced Learners' Hit Rate	Non-words
成功	cheng2gong1	*success*	0.25	0.79	*cheng1gong1
房间	fang2jian1	*room*	0.75	0.86	*fang1jian1
房租	fang2zu1	*rent*	0.58	0.64	*fang1zu1
服装	fu2zhuang1	*clothing*	0.25	0.64	*fu1zhuang1
黄瓜	huang2gua1	*cucumber*	0.92	0.71	*huang1gua1
爬山	pa2shan1	*mountain climbing*	0.67	0.86	*pa1shan1
十分	shi2fen1	*very*	0.92	1.00	*shi1fen1
时间	shi2jian1	*time*	0.67	0.79	*shi1jian1
维修	wei2xiu1	*maintain*	0.17	0.79	*wei1xiu1
阳光	yang2guang1	*sunshine*	0.25	0.86	*yang1guang1

T1-to-T3					
Real Words			Beginners' Hit Rate	Advanced Learners' Hit Rate	Non-words
冬天	dong1tian1	*winter*	0.92	1.00	*dong3tian1
关心	guan1xin1	*concern*	0.67	1.00	*guan3xin1
今天	jin1tian1	*today*	0.92	1.00	*jin3tian1
欧洲	ou1zhou1	*Europe*	1.00	1.00	*ou3zhou1
身高	shen1gao1	*height*	0.42	0.79	*shen3gao1
书桌	shu1zhuo1	*desk*	0.58	0.71	*shu3zhuo1
西装	xi1zhuang1	*suit*	0.42	0.86	*xi3zhuang1
香蕉	xiang1jiao1	*banana*	0.33	0.93	*xiang3jiao1
医生	yi1sheng1	*doctor*	1.00	1.00	*yi3sheng1
中心	zhong1xin1	*center*	1.00	1.00	*zhong3xin1

T3-to-T1					
Real Words			Beginners' Hit Rate	Advanced Learners' Hit Rate	Non-words
北京	bei3jing1	*Beijing*	1.00	1.00	*bei1jing1
饼干	bing3gan1	*cookie*	0.67	0.64	*bing1gan1
打开	da3kai1	*open*	0.75	0.93	*da1kai1
打针	da3zhen1	*injection*	0.75	0.64	*da1zhen1
果汁	guo3zhi1	*juice*	0.25	0.71	*guo1zhi1
取消	qu3xiao1	*cancel*	0.25	0.64	*qu1xiao1
手机	shou3ji1	*cell phone*	0.67	0.93	*shou1ji1
首都	shou3du1	*captital*	0.92	1.00	*shou1du1
小偷	xiao3tou1	*thief*	0.58	0.86	*xiao1tou1
已经	yi3jing1	*already*	0.83	0.93	*yi1jing1

T1-to-T4					
Real Words			Beginners' Hit Rate	Advanced Learners' Hit Rate	Non-words
搬家	ban1jia1	*move*	0.67	0.86	*ban4jia1
冰箱	bing1xiang1	*fridge*	0.50	0.86	*bing4xiang1
参加	can1jia1	*participate*	0.58	1.00	*can4jia1
公斤	gong1jin1	*kilogram*	0.50	0.71	*gong4jin1
交通	jiao1tong1	*traffic*	0.67	1.00	*jiao4tong1
沙发	sha1fa1	*sofa*	1.00	1.00	*sha4fa1
西方	xi1fang1	*west*	0.42	0.93	*xi4fang1
星期	xing1qi1	*week*	1.00	1.00	*xing4qi1
糟糕	zao1gao1	*bad*	1.00	1.00	*zao4gao1
中间	zhong1jian1	*middle*	0.58	0.93	*zhong4jian1

T4-to-T1					
Real Words			Beginners' Hit Rate	Advanced Learners' Hit Rate	Non-words
菜单	cai4dan1	*menu*	0.50	0.93	*cai1dan1
大家	da4jia1	*everyone*	0.92	0.93	*da1jia1
蛋糕	dan4gao1	*cake*	0.92	1.00	*dan1gao1
电车	dian4che1	*tram*	0.50	0.79	*dian1che1
互相	hu4xiang1	*mutual*	0.08	0.79	*hu1xiang1
客厅	ke4ting1	*living room*	1.00	0.93	*ke1ting1
上班	shang4ban1	*work*	0.75	1.00	*shang1ban1
信息	xin4xi1	*information*	0.50	0.43	*xin1xi1
亚洲	ya4zhou1	*Asia*	0.75	0.71	*ya1zhou1
再说	zai4shuo1	*what's more*	0.75	1.00	*zai1shuo1

T2-to-T3					
Real Words			Beginners' Hit Rate	Advanced Learners' Hit Rate	Non-words
回家	hui2jia1	*go home*	1.00	1.00	*hui3jia1
离开	li2kai1	*leave*	0.92	1.00	*li3kai1
聊天	liao2tian1	*chat*	0.83	0.93	*liao3tian1
骑车	qi2che1	*cycling*	0.50	0.43	*qi3che1
其中	qi2zhong1	*among*	0.25	0.86	*qi3zhong1
前天	qian2tian1	*the day before yesterday*	0.33	0.93	*qian3tian1
时差	shi2cha1	*jet lag*	0.50	0.79	*shi3cha1
职工	zhi2gong1	*staff*	0.25	0.50	*zhi3gong1
直接	zhi2jie1	*direct*	0.25	0.93	*zhi3jie1
昨天	zuo2tian1	*yesterday*	0.92	1.00	*zuo3tian1

T3-to-T2					
Real Words			Beginners' Hit Rate	Advanced Learners' Hit Rate	Non-words
打车	da3che1	*take a taxi*	0.75	1.00	*da2che1
打工	da3gong1	*work*	0.83	1.00	*da2gong1
耳机	er3ji1	*earphone*	0.67	0.57	*er2ji1
海边	hai3bian1	*beach*	0.67	0.71	*hai2bian1
好吃	hao3chi1	*delicious*	0.92	1.00	*hao2chi1
火车	huo3che1	*Train*	0.83	0.86	*huo2che1
纽约	niu3yue1	*New York*	0.83	0.86	*niu2yue1
普通	pu3tong1	*ordinary*	0.42	0.93	*pu2tong1
小心	xiao3xin1	*be careful*	0.67	1.00	*xiao2xin1
有关	you3guan1	*relative*	0.25	0.79	*you2guan1

T2-to-T4					
Real Words			Beginners' Hit Rate	Advanced Learners' Hit Rate	Non-words
读书	du2shu1	*reading*	1.00	1.00	*du4shu1
黄金	huang2jin1	*gold*	0.25	0.93	*huang4jin1
结婚	jie2hun1	*get married*	0.75	0.93	*jie4hun1
离婚	li2hun1	*divorce*	0.42	1.00	*li4hun1
明天	ming2tian1	*tomorrow*	0.92	0.93	*ming4tian1
南京	nan2jing1	*Nanjing*	0.92	0.86	*nan4jing1
年轻	nian2qing1	*young*	0.42	0.93	*nian4qing1
旁边	pang2bian1	*next to*	0.92	1.00	*pang4bian1
十八	shi2ba1	*eighteen*	0.83	0.93	*shi4ba1
提高	ti2gao1	*improve*	0.92	1.00	*ti4gao1

T4-to-T2					
Real Words			Beginners' Hit Rate	Advanced Learners' Hit Rate	Non-words
衬衫	chen4shan1	*shirt*	0.67	0.43	*chen2shan1
大哥	da4ge1	*big brother*	0.75	1.00	*da2ge1
大约	da4yue1	*about*	0.33	0.86	*da2yue1
第三	di4san1	*third*	0.92	0.93	*di2san1
录音	lu4yin1	*sound recording*	0.83	0.93	*lu2yin1
念书	nian4shu1	*study*	0.92	1.00	*nian2shu1
认真	ren4zhen1	*serious*	0.83	1.00	*ren2zhen1
下车	xia4che1	*get off*	0.75	1.00	*xia2che1
夏天	xia4tian1	*summer*	0.83	0.79	*xia2tian1
现金	xian4jin1	*cash*	0.33	0.64	*xian2jin1

T3-to-T4					
Real Words			Beginners' Hit Rate	Advanced Learners' Hit Rate	Non-words
海关	hai3guan1	*customs*	0.58	1.00	*hai4guan1
好听	hao3ting1	*pleasant to hear*	1.00	1.00	*hao4ting1
简单	jian3dan1	*simple*	0.50	1.00	*jian4dan1
紧张	jin3zhang1	*nervous*	0.75	1.00	*jin4zhang1
酒吧	jiu3ba1	*bar*	0.50	0.93	*jiu4ba1
老师	lao3shi1	*teather*	1.00	1.00	*lao4shi1
起飞	qi3fei1	*take off*	0.67	0.71	*qi4fei1
小说	xiao3shuo1	*novel*	0.83	1.00	*xiao4shuo1
许多	xu3duo1	*many*	0.33	0.93	*xu4duo1
转机	zhuan3ji1	*transfer*	0.33	0.79	*zhuan4ji1

T4-to-T3					
Real Words			Beginners' Hit Rate	Advanced Learners' Hit Rate	Non-words
半天	ban4tian1	*quite a while*	0.83	0.86	*ban3tian1
唱歌	chang4ge1	*sing*	1.00	1.00	*chang3ge1
大衣	da4yi1	*coat*	0.58	0.57	*da3yi1
更加	geng4jia1	*more*	0.25	0.79	*geng3jia1
健康	jian4kang1	*health*	0.83	1.00	*jian3kang1
律师	lv4shi1	*lawyer*	0.92	0.50	*lv3shi1
面包	mian4bao1	*bread*	0.75	1.00	*mian3bao1
汽车	qi4che1	*car*	0.75	0.93	*qi3che1
用功	yong4gong1	*hardworking*	0.92	0.64	*yong3gong1
战争	zhan4zheng1	*war*	0.25	0.79	*zhan3zheng1

Segmental pairs							Segmental pairs						
Real Words			Beginners' Hit Rate	Advanced Learners' Hit Rate	Non-words		Real Words			Beginners' Hit Rate	Advanced Learners' Hit Rate	Non-words
超级	chao1ji2	*super*	0.67	0.64	*shao1ji2		打球	da3qiu2	*play ball games*	0.83	0.86	*ta3qiu2
刚才	gang1cai2	*just now*	1.00	0.86	*kang1cai2		打折	da3zhe2	*give a discount*	0.75	0.86	*ta3zhe2
高楼	gao1lou2	*high-rise*	0.75	1.00	*hao1lou2		海牙	hai3ya2	*Den Haag*	1.00	0.93	*kai3ya2
公园	gong1yuan2	*park*	1.00	1.00	*hong1yuan2		检查	jian3cha2	*check*	0.58	0.79	*xian3cha2
忽然	hu1ran2	*suddenly*	0.75	0.86	*ku1ran2		解决	jie3jue2	*solve*	0.75	0.93	*xie3jue2
开门	kai1men2	*open the door*	1.00	1.00	*gai1men2		警察	jing3cha2	*policeman*	0.50	0.86	*xing3cha2
商人	shang1ren2	*businessman*	0.58	0.79	*zhang1ren2		旅行	lv3xing2	*trip*	1.00	1.00	*nv3xing2
生活	sheng1huo2	*life*	0.67	0.93	*cheng1huo2		美元	mei3yuan2	*dollar*	0.58	0.57	*bei3yuan2
中国	zhong1guo2	*China*	1.00	0.86	*chong1guo2		奶油	nai3you2	*cream*	0.92	1.00	*dai3you2
中文	zhong1wen2	*Chinese*	1.00	0.93	*chong1wen2		小时	xiao3shi2	*hour*	1.00	0.86	*jiao3shi2
成为	cheng2wei2	*become*	0.75	1.00	*sheng2wei2		大学	da4xue2	*university*	1.00	0.93	*ta4xue2
厨房	chu2fang2	*kitchen*	0.67	0.79	*shu2fang2		地图	di4tu2	*map*	1.00	1.00	*ti4tu2
荷兰	he2lan2	*Netherlands*	1.00	1.00	*ke2lan2		进来	jin4lai2	*come in*	0.75	0.93	*xin4lai2
回答	hui2da2	*answer*	0.83	1.00	*kui2da2		客人	ke4ren2	*guest*	0.83	1.00	*he4ren2
零食	ling2shi2	*snack*	0.67	0.86	*ning2shi2		课文	ke4wen2	*text*	0.92	0.93	*ge4wen2
前年	qian2nian2	*the year before last year*	0.75	0.86	*xian2nian2		气球	qi4qiu2	*balloon*	0.33	0.43	*ji4qiu2
实习	shi2xi2	*internship*	0.50	0.86	*chi2xi2		去年	qu4nian2	*last year*	0.83	1.00	*ju4nian2
学习	xue2xi2	*study*	1.00	1.00	*jue2xi2		认为	ren4wei2	*think*	0.83	0.93	*zhen4wei2
着急	zhao2ji2	*worry*	0.42	0.79	*shao2ji2		上楼	shang4lou2	*go upstairs*	0.92	0.93	*chang4lou2
足球	zu2qiu2	*football*	0.92	0.93	*su2qiu2		适合	shi4he2	*suitable*	0.83	0.93	*chi4he2
